# ARF1 and GBF1 Generate a PI4P-Enriched Environment Supportive of Hepatitis C Virus Replication

**DOI:** 10.1371/journal.pone.0032135

**Published:** 2012-02-16

**Authors:** Leiliang Zhang, Zhi Hong, Wenyu Lin, Run-Xuan Shao, Kaku Goto, Victor W. Hsu, Raymond T. Chung

**Affiliations:** 1 Institute of Pathogen Biology, Chinese Academy of Medical Sciences and Peking Union Medical College, Beijing, China; 2 Gastrointestinal Unit, Department of Medicine, Massachusetts General Hospital, Harvard Medical School, Boston, Massachusetts, United States of America; 3 Division of Rheumatology, Immunology, and Allergy, Brigham and Women's Hospital, and Department of Medicine, Harvard Medical School, Boston, Massachusetts, United States of America; University of Kansas Medical Center, United States of America

## Abstract

Cellular levels of phosphatidylinositol 4-phosphate (PI4P) have been shown to be upregulated during RNA replication of several viruses, including the HCV replicon model. However, whether PI4P is required in an infectious HCV model remains unknown. Moreover, it is not established whether the host transport machinery is sequestered by the generation of PI4P during HCV infection. Here we found that PI4P was enriched in HCV replication complexes when Huh7.5.1 cells were infected with JFH1. HCV replication was inhibited upon overexpression of the PI4P phosphatase Sac1. The PI4P kinase PI4KIIIβ was also found to be required for HCV replication. Moreover, the vesicular transport proteins ARF1 and GBF1 colocalized with PI4KIIIβ and were both required for HCV replication. During authentic HCV infection, PI4P plays an integral role in virus replication.

## Introduction

Hepatitis C virus (HCV), a positive-strand RNA virus of the family Flaviviridae, is one of the most prominent health threats in the world today, infecting more than 170 million persons globally [1]. Current HCV treatment with pegylated interferon α and ribavirin. produces sustained virologic response (SVR) in 42–52%, 65–85% and 76–82% of individuals infected with HCV genotype 1, HCV genotypes 4, 5 or 6 and HCV genotypes 2 or 3, respectively [2], [3]. Many inhibitors targeting HCV viral proteins have been discovered with many of them at late stages of clinical trials. Currently several new classes of antiviral drugs targeting NS5A and NS5B are at various stages of preclinical and clinical studies [4]. In 2011 two NS3 protease inhibitors, telaprevir and boceprevir, have been approved. An enhanced sustained virologic response (SVR) can be achieved through addition of protease inhibitors. Recent trials in patients with HCV genotype 1 infection combining the standard of care (SOC) with telaprevir or boceprevir have found that SVR rates is 61–75% for telaprevir [5], [6], [7], [8] or 67–75% for boceprevir [9], [10], [11]. While new agents directly targeting HCV proteins have been developed, they are limited by the selection of resistant variants. Understanding the cell biology of viral replication is critical for the development of novel host-directed antiviral strategies against HCV that could circumvent the selection of resistant viral variants.

The 9.6 kb genome of HCV encodes structural proteins including core, E1 and E2 and the nonstructural proteins consisting p7, NS2–NS5 which support viral RNA replication. HCV displays a remarkable capacity to survive and replicate in its host [12]. To be successful in establishing persistent infection, HCV hijacks host cellular processes at several levels. Among these disrupted processes are lipid signaling and metabolism [13], [14], [15], [16], which provide a novel insight into host-pathogen interactions.

As a key member of the phosphatidylinositol (PI), phosphatidylinositol 4-phosphate (PI4P) is the principle phosphoinositide in the Golgi apparatus and acts as a targeting signal for Golgi associated proteins [17]. PtdIns 4-kinases (PI4K) phosphorylate PI at the 4′ position to yield PI4P. Eukaryotes contain two classes of PtdIns 4-kinase, termed type II and type III. Type II PtdIns 4-kinases make up a significant fraction of the PtdIns 4-kinase activities in plasma membrane, whereas type III PtdIns 4-kinases, namely PI4KIIIα and PI4KIIIβ, serve to generate pools of PI4P in Golgi [18]. Sac1, a type II membrane protein that localizes to the ER and Golgi apparatus, is a principle pathway for PI(4)P dephosphorylation in vivo [19]. Investigating the mechanisms by which pathogens exploit PI4P could not only identify potential new antiviral targets but will also reveal additional insights into the cellular function of PI4P.

Recently, Hsu et al [20] found that PI4P is involved in HCV replication in a replicon model and that the distribution of PI4P changes in cells that harbor stable viral polyproteins with a selectable marker. Studies from several groups demonstrated that NS5A recruited PI4KIIIα to the viral replication compartment and stimulated PI4KIIIα activity for HCV replication [21], [22], [23]. In this study, we assessed whether PI4P is similarly involved in HCV replication in the fully infectious JFH1 model. Moreover, we found that two vesicular transport proteins, ARF1 and GBF1, colocalized with PI4KIIIβ, and that both proteins were required for HCV replication.

## Results

### PI4P is rearranged during HCV infection

**Figure 1:**
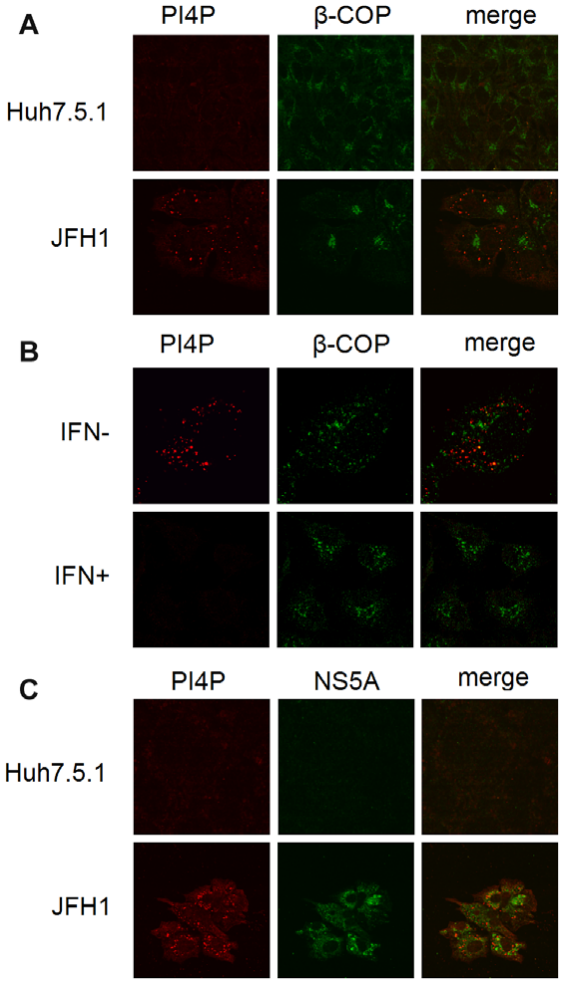
PI4P is rearranged during HCV infection. (A) Huh 7.5.1 cells infected or uninfected with JFH1 were stained with antibodies against PI4P (red) or β-COP (green). (B) Huh 7.5.1 cells infected with JFH1 were treated with 90 IU/ml IFN or mock infection and then stained with antibodies against PI4P (red) or β-COP (green). (C) Huh 7.5.1 cells infected or uninfected with JFH1 were stained with antibodies against PI4P (red) or NS-5A (green).

To assess the localization of PI4P during HCV infection, Huh 7.5.1 cells were infected with the cell culture infectious JFH1 for 3 days followed by immunofluorescence microscopy. As shown in Figure 1A, PI4P staining in JFH1 infected-Huh7.5.1 (JFH1) cells was significantly stronger than that in the mock infected cells (Figure 1A). The β–COP is a Golgi marker. To validate that upregulation of PI4P relied on HCV replication, we infected Huh7.5.1 cells with JFH1 in the presence of 90 IU/ml IFN to repress HCV replication and found that the PI4P signal is reduced by IFN (Figure 1B), Moreover, PI4P partially colocalized with NS5A in the JFH1 model (Figure 1C). Taken together, PI4P is rearranged during HCV infection and could define the viral replication compartment.

### Sac1 overexpression inhibits HCV replication

**Figure 2:**
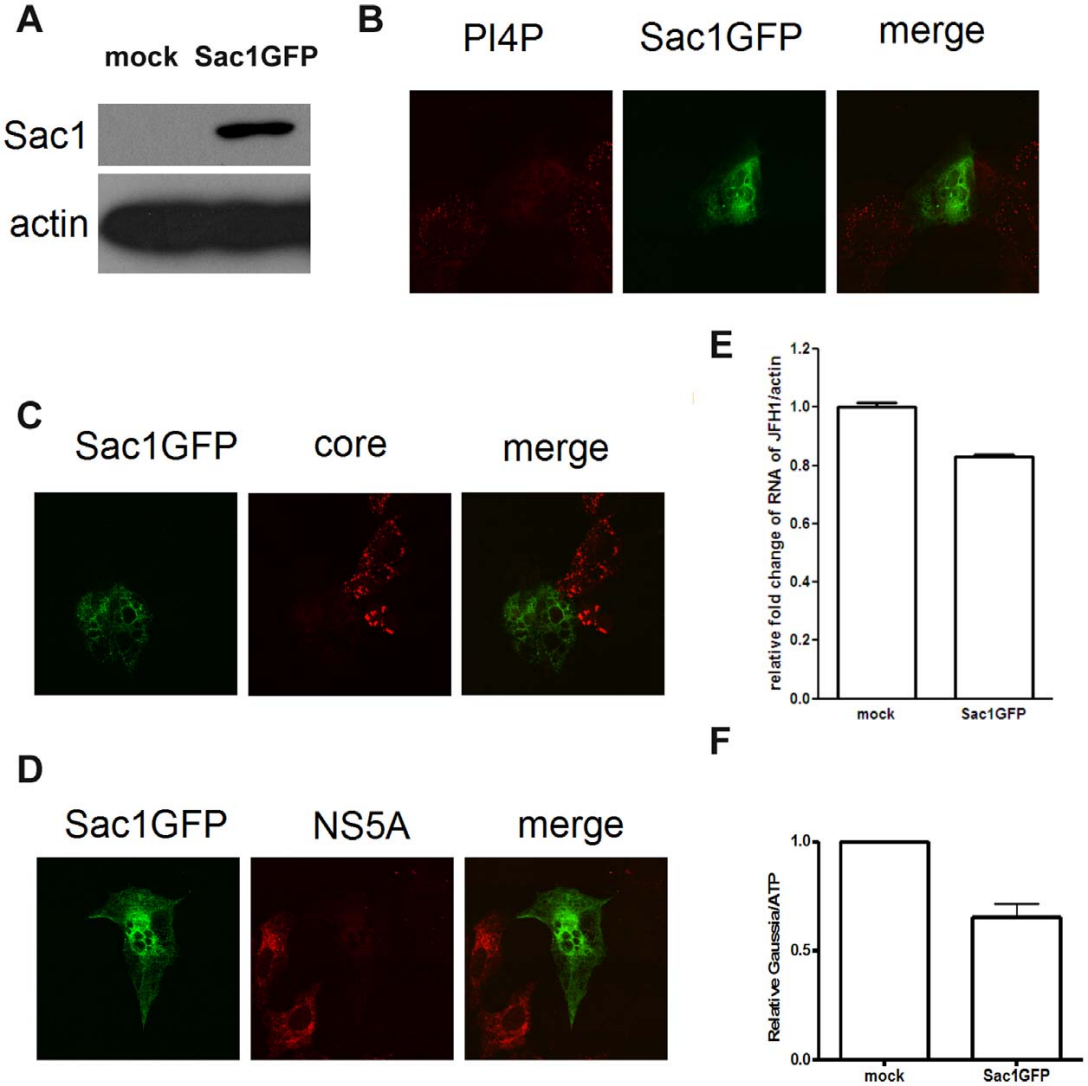
Sac1 overexpression inhibits HCV replication. (A) Huh 7.5.1 cells transfected with pEGFP-Sac1 or mock and then cell lysates were analyzed by immunoblotting with the indicated antibodies. (B) Huh 7.5.1 cells transfected with pEGFP-Sac1 and infected with JFH1 were stained with antibodies against PI4P (red). (C) Huh 7.5.1 cells transfected with pEGFP-Sac1 and infected with JFH1 were stained with antibodies against HCV core (red). (D) Huh 7.5.1 cells transfected with pEGFP-Sac1 and infected with JFH1 were stained with antibodies against HCV NS5A (red). (E) Huh 7.5.1 cells were transfected with pEGFP-Sac1 and infected with JFH1. Total RNA was isolated and reversely transcribed and then quantitive PCR was performed. (F) Huh7.5.1 cells were transfected with pEGFP-Sac1 and infected with Jc1FLAG2(p7-nsGluc2A) for 3 days. Gaussia luciferase activity and cellular ATP levels were measured. The normalized luciferase activities were then divided by the normalized luciferase activity from mock treatment.

Sac1 is a phosphoinositide lipid phosphatase that removes the phosphate residue from the inositol head group of PI(4)P [24]. To explore the importance of PI4P in HCV replication, we overexpressed Sac1 and then infected Huh7.5.1 cells with JFH1. The expression of Sac1GFP was confirmed by western blot (Figure 2A) and cellular PI4P was depleted by Sac1GFP (Figure 2B). As shown in Figure 2C and 2D, HCV replication as measured by HCV core and NS5A staining was strongly inhibited by overexpression of Sac1. To further establish the effect of Sac1 overexpression on HCV replication, we infected Huh7.5.1 cells with JFH1 and total RNA was isolated for reverse transcription and subsequent quantitive PCR ana lysis. We observed modest reduction of actin-normalized JFH1 RNA levelswith overexpression of Sac1GFP of about 20% (Figure 2E), but this appears to have been correlated with reduced efficiency of Sac1 transfection in Huh7.5.1 cells. Next Huh7.5.1 cells were transfected with pEGFP-Sac1 and infected with Jc1FLAG2(p7-nsGluc2A) for 3 days. Gaussia luciferase activity and cellular ATP levels were measured. The normalized luciferase activities were then divided by the normalized luciferase activity from mock treatment. The reduction of Jc1 measured by lucifease activity by overexpression of Sac1GFP is only about 35% (Figure 2F). These data demonstrated the role of Sac1 for HCV replication and confirm an essential role for PI4P in HCV replication.

### PI4KIIIβ is required for HCV replication

**Figure 3:**
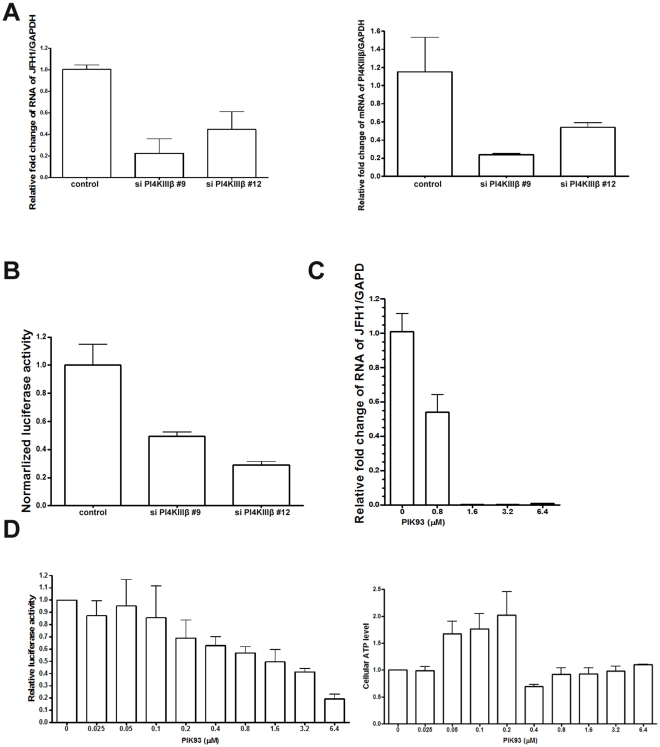
PI4KIIIβ is required for HCV replication. (A) Huh7.5.1 cells were treated with siRNA against PI4KIIIβ or control siRNA for 3 days and then infected with JFH1 for 3 days. Total RNA was isolated and reversely transcribed and then quantitive PCR was performed. (B) Huh7.5.1 cells were treated with siRNA against PI4KIIIβ or control siRNA for 3 days and then infected with Jc1 for 3 days. Gaussia luciferase activity and cellular ATP levels were measured. The normalized luciferase activities were then divided by the normalized luciferase activity from mock treatment. (C) Huh7.5.1 cells were treated with different dosages of PIK93 as indicated for 1 hour and then infected with JFH1 for 3 days. Total RNA was isolated and reversely transcribed and then quantitive PCR was performed. (D) Huh7.5.1 cells were treated with different dosages of PIK93 as indicated for 1 hour and then infected with Jc1FLAG2(p7-nsGluc2A) for 3 days. Gaussia luciferase activity and cellular ATP levels were measured. The normalized luciferase activities were then divided by the normalized luciferase activity from mock treatment.

To gain insight into the importance of PI4P levels regulated by PI4P kinases, we knocked down the PI4P kinase PI4KIIIβ in Huh7.5.1 cells and infected these cells with JFH1. As Figure 3A shows, RNA replication of JFH1 is reduced upon knock down of PI4KIIIβ by siRNA. To further confirm the role of PI4KIIIβ, we knocked down PI4KIIIβ in uninfected Huh7.5.1 cells and cells infected with the infectious virus Jc1FLAG2(p7-nsGluc2A). We found that the replication of Jc1FLAG2(p7-nsGluc2A) as measured by luciferase activity is markedly reduced with PI4KIIIβ knockdown (Figure 3B). PIK93 is an inhibitor of PI4KIIIβ [25]. We treated Huh7.5.1 cells with increasing doses of PIK93 and infected with either JFH1 or Jc1FLAG2(p7-nsGluc2A) . PIK93 did not reduce cell viability as measured by cellular ATP levels even at doses of 6.4 µM (Figure 3D). At a dosage of 0.8 µM, PIK93 inhibited HCV replication by around 45% as assessed by QPCR (Figure 3C) or luciferase activity (Figure 3D). Taken together, PI4KIIIβ is required for HCV replication.

### PI4KIIIβ colocalizes with ARF1 or GBF1

**Figure 4:**
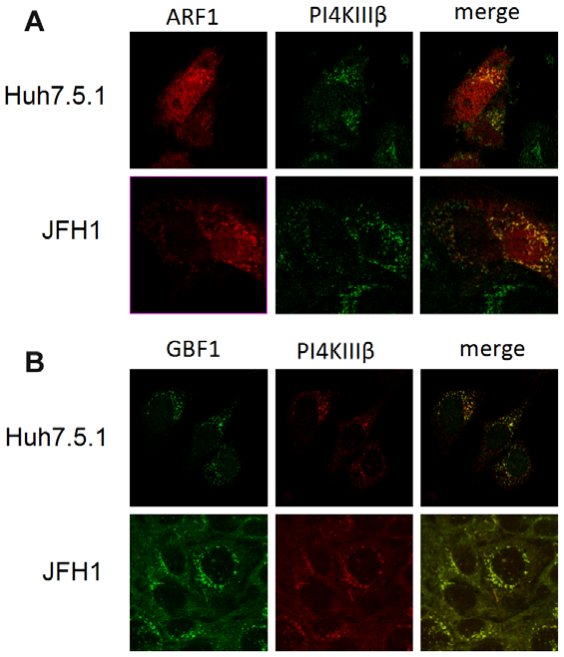
PI4KIIIβ colocalizes with ARF1 or GBF1. (A) Huh 7.5.1 cells transfected with a HA-tagged ARF1 construct and infected with JFH1 or mock were stained with antibodies against HA (red) or PI4KIIIβ (green). (B) Huh 7.5.1 cells infected with JFH1 or mock were stained with antibodies against PI4KIIIβ (red) or GBF1 (green).

Activated ARF1 is responsible for the delivery of PI4KIIIβ to the Golgi membrane where the local concentration of PI4P is enriched [26]. We examined whether ARF1 colocalized with PI4KIIIβ during HCV infection. We transfected an HA tagged ARF1 plasmid to Huh7.5.1 cells and infected them with JFH1 or mock. We found that ARF1 colocalized with PI4KIIIβ (Figure 4A). We also found that one of the GEFs (guanine nucleotide exchange factors) for ARF1, GBF1, colocalized with PI4KIIIβ (Figure 4B). These data suggest that GBF1 and ARF1 generate sufficient local concentrations of PI4P to enable HCV infection.

### ARF1 is required for HCV replication

**Figure 5:**
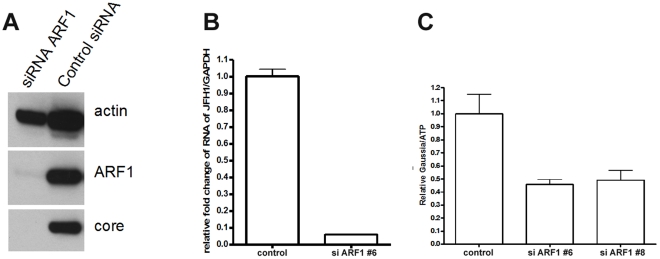
ARF1 is required for HCV replication. (A) Huh7.5.1 cells were treated with siRNA against PI4KIIIβ or control siRNA for 3 days and then infected with JFH1 for 3 days. Cell lysates were analyzed by immunoblotting with the indicated antibodies. (B) Huh7.5.1 cells were treated with siRNA against ARF1 or control siRNA for 3 days and then infected with JFH1 for 3 days. Total RNA was isolated and reverse transcribed and then quantitive PCR was performed. (C) Huh7.5.1 cells were treated with siRNA against ARF1(#6 sequence or #8 sequence) or control siRNA and then infected with Jc1FLAG2(p7-nsGluc2A) for 3 days. Gaussia luciferase activity and cellular ATP levels were measured. The normalized luciferase activities were then divided by the normalized luciferase activity from mock treatment.

Next we investigated the effect of ARF1 inhibition on HCV replication. When Huh7.5.1 cells were knocked down ARF1 by siRNA (Figure 5A and 5B) and infected with JFH1, HCV replication was reduced 10-fold as assessed by western blot (Figure 5A) and QPCR (Figure 5B). Next Huh7.5.1 cells were treated with two individual siRNAs against ARF1 (#6 and #8) and infected with Jc1FLAG2(p7-nsGluc2A) for 3 days. Gaussia luciferase activity and cellular ATP levels were measured. The normalized luciferase activities were then divided by the normalized luciferase activity from mock treatment. Both siRNAs could strongly reduce HCV replication (Figure 5C).These data indicate that ARF1 is required for HCV replication.

### GBF1 is required for HCV replication

**Figure 6:**
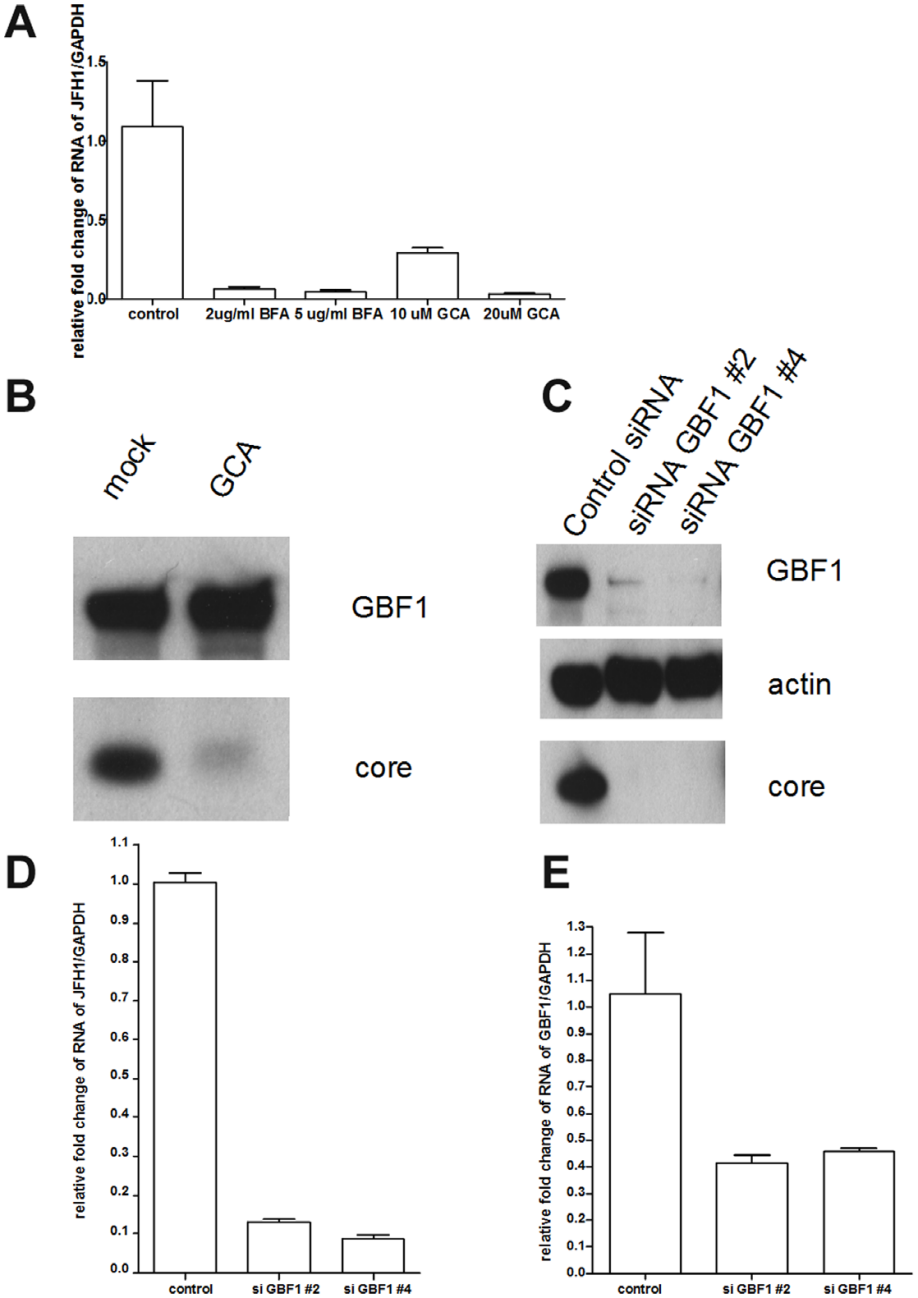
GBF1 is required for HCV replication. (A) Huh7.5.1 cells were treated with different dosages of BFA or GCA as indicated for 1 hour and then infected with JFH1 for 2 days. Total RNA was isolated and reverse transcribed and then quantitive PCR was performed. (B) Huh7.5.1 cells were treated with 20 µM GCA for 1 hour and then infected with JFH1 for 3 days. Cell lysates were analyzed by immunoblotting with the indicated antibodies. (C) Huh7.5.1 cells were treated with siRNA against GBF1 or control siRNA for 3 days and then infected with JFH1 for 3 days. Cell lysates were analyzed by immunoblotting with the indicated antibodies. (D) Huh7.5.1 cells were treated with siRNA against GBF1 or control siRNA for 3 days and then infected with JFH1 for 3 days. Total RNA was isolated and reverse transcribed and then quantitive PCR was performed.

To examine whether GBF1 is critical for HCV replication, Huh7.5.1 cells were treated with the GBF1 inhibitor GCA or ARF1 inhibitor BFA, infected with JFH1, and assessed for HCV replication. Both BFA and GCA reduced HCV RNA replication up to 10-fold as shown in Figure 6A. Morevoer HCV core protein level was reduced by GCA (Figure 6B). Two siRNAs against GBF1 also inhibited HCV replication (Figure 6C and 6D). These data confirm that GBF1 is required for HCV replication.

## Discussion

Inositol phospholipids play important regulatory roles in a variety of cellular processes. In total, seven species of phosphoinositides are generated by the reversible phosphorylation of the inositol headgroups, which mediate the specific lipid-protein interactions on membrane-cytosol interfaces [27]. Each of the seven phosphoinositides has a unique spatial distribution with a predominant localization in subcellular organelles that define their identities. For example, PI(4,5)P2 is mainly found in the plasma membrane, PI3P in endosomes and phagosomes, PI(3,5)P2 in vacuoles and late endosomes, while PI4P is enriched in the Golgi complex region [17], [27]. The differential intracellular distribution of phosphoinositides, together with their high turnover rate makes these lipids optimal regulators for precise intracellular activities, such as signaling transduction, cytoskeleton organization and membrane dynamics.

Investigation of PI metabolism perturbed by HCV may provide significant opportunity to understand the regulation of PI during viral infection, identifying specific PI effectors for further dissection of their functions and assessment as plausible therapeutic candidates. Here we found that PI4P is enriched in HCV replication complexes. In the report of Hsu [20], NS5A clearly colocalizedwith PI4P in the HCV replicon cell. In contrast, in our study, PI4P only partially colocalized with NS5A in the JFH1 model (Figure 1B), suggesting that the upregulation and rearrangement of PI4P during authentic HCV infection differs from that of the replicon model, which invariably overexpresses viral proteins.

To understand the spatial and temporal restriction of PI4P during virus infection, it becomes necessary to identify the phosphoinositide kinases and phosphatases involved in PI4P homeostasis and their regulation. Sac1 is an ER- and Golgi-complex-associated phosphoinositide phosphatase that dephosphorylates PI4P during retrograde transport from Golgi to ER, thus ensuring the enrichment of PI4P in the Golgi complex [19], [24]. When we expressed Sac1 to deplete PI4P, HCV replication was repressed, which suggests that PI4P is crucial for HCV replication.

The importance of PI4P in Golgi structure and function was revealed by mutations of the PI4P kinase Pik1, which resulted in an accumulation of abnormal membrane intermediates with defect of transport to plasma membrane [17]. The mammalian Pik1 homolugue is PI4KIIIβ. However, PI4KIIIα has been identified by several RNAi screens as a host factor for HCV replication [21], [28], [29], [30], [31], [32]. NS5A is able to recruit PI4KIIIα to the viral replication compartment and stimulate PI4KIIIα activity, which is crucial for HCV replication [21], [22], [23]. Thus it is well established that PI4P generated by PI4KIIIα is important for HCV replication. In other studies, PI4KIIIβ was identified as the kinase responsible for generating PI4P during HCV replication [20], [33]. Here, we confirm that PI4KIIIβ is critical for HCV replication in JFH1 and Jc1 infection models. Nonetheless, it remains possible that both PI4KIIIα and PI4KIIIβ are required for HCV replication.

Beside phosphoinositides, another class of molecules mediated the recruitment of cytosolic protein to specific membrane compartment is small GTPases [34]. They shuttle between GTP (active) and GDP (inactive) bound states with the help of Guanidine nucleotide exchange factors (GEFs) and GTPase activating proteins (GAP), which stimulate the GTP loading and hydrolysis respectively. ARF1 regulates the vesicular transport from Golgi to ER [34]. It has been shown that ARF1 promotes the production of PI4P within the Golgi membrane by recruiting PI4KIIIβ [26]. Mounting functional and mechanistic evidence suggests that components of the COPI pathway act as host lipid effectors for a number of pathogens, such as poliovirus [35], [36], [37], HIV [35] and vaccinia virus [38]. Investigating the mechanisms by which viruses exploit COPI, as well as the mechanisms by which they inhibit COPI, will not only identify possible new antiviral targets but will also reveal additional insight into COPI normal cellular function. In this study we found that during HCV infection, ARF1 and its exchange factor GBF1 colocalized with PI4KIIIβ on the HCV replication complex. Recently, we conducted an RNAi screen and identified the core factor of COPI pathway coatomer as a host factors involved in HCV replication [30]. Other two groups found that key COPI pathway factors, including GBF1 and ARF1 are critical for HCV replication [39], [40]. Here we also show that ARF1 and GBF1 are required for HCV replication, further establishing that COPI pathway proteins are pro-viral factors for HCV. Moreover, we propose that one of the mechanisms of COPI participating in the HCV replication is to divert PI4KIIIβ from the Golgi complex to the viral replication compartment to generate a new PI4P enriched environment. Further work is necessary to identify viral proteins that are involved in targeting PI4KIIIβ, ARF1 and GBF1 to the replication complex.

During the lifecycle of HCV, many host cellular processes are subverted. For instance, lipid droplets are crucial for the formation of infectious HCV virus particles [16]. A number of host and viral proteins are recruited to the HCV replication complex. The accuracy of protein targeting may depend on the restriction and the generation of PI4P in this replication organelle. Several PI4P binding proteins such as OSBP1 [41] and CERT [42] that contains PI4P binding motif have been identified as host factors for HCV replication. OSBP1 and CERT can recognize PI4P by lipid-specific targeting domains to mediate organelle-specific recruitment. Further investigations of more host and viral PI4P associated proteins are required to elucidate the detailed mechanism of the role of PI4P in HCV replication.

The HCV transmembrane protein NS4B is able to induce the membranous web and could recruit the polymerase complex to the HCV RNA. Great efforts have been made to identify pharmacological inhibitors of NS4B [43]. However whether PI4P is involved in membranous web formation or not is unknown; further study is are needed.

In conclusion, during authentic HCV infection, PI4P plays an integral role in virus replication. It is synthesized in the RNA replication complex by PI4KIIIβ, which is recruited specifically to the replication complex by host transport proteins GBF1 and ARF1. Our current findings suggest that the blockade of PI4P formation is a potentially novel, host-directed antiviral target that could complement more conventional antiviral therapy directed at viral protein targets.

## Materials and Methods

### Cells, virus and reagents

Huh7.5.1 cells were grown in Dulbecco's Modified Eagle's Medium (DMEM) supplemented with 10% fetal bovine serum (FBS). Infectious JFH1 plasmid was obtained from Dr. Takaji Wakita and inoculated as previously described [44]. The infectious plasmid Jc1FLAG2(p7-nsGluc2A) expressing *Gaussia* luciferase was obtained from Dr. Charles Rice [45]. The PI4KIIIβ inhibitor PIK93 was obtained from Symansis (Auckland, New Zealand). BFA and GCA were obtained from Sigma Life Science and Biochemicals (St. Louis, MO.). Construct pEGFP-Sac1 was gift from Dr. Peter Mayinger [46].

### Western blotting

Cells were lysed using radioimmune precipitation assay (RIPA) buffer containing 1% NP-40, 0.1% SDS, 10 mM Tris-HCl (pH 7.4), 1 mM EDTA, 150 mM NaCl and protease inhibitor cocktail and subsequently sonicated. Proteins were separated by SDS-PAGE with NuPAGE Novex pre-cast 4–12% Bis-Tris gradient gels (Invitrogen, Carlsbad, CA) and transferred to PVDF membranes. The primary antibodies used including mouse anti-GBF1 (BD Biosciences), mouse anti-ARF1 (Novus Biologicals, Littleton, CO), mouse anti-HCV core (Affinity BioReagents Inc., Golden, CO), mouse anti-actin (Sigma Life Science and Biochemicals, St. Louis, MO). Secondary antibodies were HRP-conjugated ECL donkey anti-rabbit IgG and HRP-conjugated ECL sheep anti-mouse IgG (Amersham Biosciences, Piscataway, NJ). The ECL Western Blotting Detection Kit (Amersham Biosciences, Piscataway, NJ) was used to detect Chemiluminescent signals.

### Immunofluorescence microscopy

Huh7.5.1 or JFH1 cells were fixed with 4% paraformaldehyde at RT, permeabilized using 0.5% TritonX-100, and blocked with 3% BSA in PBS. The primary antibodies used in this paper were mouse anti-HCV core (ViroGen Co., Watertown, MA), NS5A (Virogen, Watertown, MA), mous anti-PI4P (Echelon Biosciences, Salk Lake City, UT), rabbit anti-β-COP (Abcam, Cambridge, MA), mouse anti-GBF1 (BD Biosciences), mouse anti-HA (Cell Signaling Technology, Danvers, MA), rabbit anti-PI4KIIIβ (Millipore). The secondary antibody was goat anti-mouse-Alexa Fluor 488 (Invitrogen, Carlsbad, CA). Immunofluorescence was observed using Nikon Eclipse 800 microscopy with the Bio-Rad Radiance 2000 confocal fluorescence microscope system (Bio-Rad Laboratories, Hercules, CA).

### Luciferase Assay

HCV replication in Jc1FLAG2(p7-nsGluc2A)-infected Huh 7.5.1 cells was determined by monitoring *Gaussia* luciferase activity (Promega, Madison, WI).

### siRNA and transfection

Indicated siRNAs were transfected into cells using Lipofectamine™ RNAiMAX Transfection Reagent (Invitrogen, Carlsbad, CA). Negative control siRNA was from QIAGEN. All siRNAs used for gene knock-down were from Dharmacon and were as follows: PI4KIIIβ, Dharmacon J-006777-09 (CCUUUAAGCUGACCACAGA)(#9), J-006777-12 (GGACUCACCAGCGCUCUAA) (#12); ARF1 Dharmacon D-011580-06 (CGGCCGAGAUCACAGACAA)(#6); D-011580-08 (GAACCAGAAGUGAACGCGA)(#8); GBF1 Dharmacon J-019783-06 (CAACACACCUACUAUCUCU), J-019783-08 (CCACUGCUGUCACU CUCUA). The protein expression of each gene knock down was confirmed by Western blotting or QPCR.

### Cell Viability Assay

Cell viability was monitored using the Cell Titer-Glo Luminescent Cell Viability Assay Kit (Promega, Madison, WI) according to the manufacturer's protocol.

### Quantitative PCR (qPCR)

Total cellular and viral RNA was isolated post infection using RNeasy Mini columns (QIAGEN) and reverse transcribed by random priming with the High Capacity cDNA Reverse Transcription Kit (Applied Biosystems; Foster City, CA), then quantified by real-time PCR using the DyNAmo HS SYBR Green qPCR kit (Finnzyme; Espoo, Finland). The primers are listed in Table 1.

**Table 1:** Primers used for quantitative RT-PCR.

Target gene	Primer^a^	Nucleotide sequence
GAPDH	F	5′-CAACTGGTCGTGGACAACCAT-3′
	R	5′-GCACGGACACTCACAATGTTC-3′
Actin	F	5′-GCACTCTTCCAGCCTTCCT-3′
	R	5′-AGGTCTTTGCGGATGTCCAC-3′
ARF1	F	5′- GTGACCACCATTCCCACCATAG-3′
	R	5′- TCATTGCTGTCCACCACGAAG-3′
GBF1	F	5′- GGGAACGCATTGACTGTTTT-3′
	R	5′- CTCGGGCTTCTCAAAGTCAC-3′
PI4KIIIβ	F	5′- TTCTTCAGACATGCACATTTCCA -3′
	R	5′- CAGTCGAACAGGCTCATCCTC-3′
JFH1	F	5′- TCTGCGGAACCGGTGAGTA-3′
	R	5′-TCAGGCAGTACCACAAGGC-3′

a F, forward; R, reverse.

### Statistics

Data analysis was performed using a 2-tailed Student's t-test. Data are expressed as mean ± SD of at least three sample replicates, unless stated otherwise.
